# Unveiling the role of supply chain parameters approved by blockchain technology towards firm performance through trust: The moderating role of government support

**DOI:** 10.1016/j.heliyon.2023.e21831

**Published:** 2023-11-04

**Authors:** Muhammad Farrukh Shahzad, Shuo Xu, Rimsha Baheer, Waleed Ahmad

**Affiliations:** a College of Economics and Management, Beijing University of Technology, Beijing, 100124, PR China; b Institute of Business & Management, University of Engineering and Technology, Lahore, 54000, Pakistan

**Keywords:** Supply chain alignment, Firm performance, Trust, Supply chain agility, Government support, Supply chain adaptability, PLS-SEM

## Abstract

This research study aims to reveal the role of supply chain parameters approved by blockchain technology toward firm performance through trust. This study has also examined the moderating role of government support between trust and firm performance. The underlying theories reinforce the usefulness and flexibility of a supply chain in regulating daily fluctuations and uncertainties in supply and demand. Blockchain technology adoption with supply chain tactics provides a more suitable environment for better firm performance. Governments address legal and security concerns related to blockchain technology and provide clear guidelines and standards for its use in supply chains, which build trust among firms and stakeholders. A conceptual model has been developed with the assistance of past empirical research studies and associated theories. This research study has examined the following relationships from a sample of 465 employees from textile industrial firms listed on the Pakistan Stock Exchange. The current research study assesses these parameters using the partial least squares structural equation modeling (PLS-SEM) method. The analysis showed that supply chain parameters (alignment, agility, adaptability) approved by blockchain technology positively correlate with firm performance. Trust positively mediated the relationship between supply chain parameters approved by blockchain technology and firm performance. Furthermore, government support positively moderated the relationship between trust and firm performance. The study would provide directions for further research. These findings will give the global supply chain industry valuable insights into blockchain technology for firm performance. In theory, this research study would contribute to the scientific literature by answering how trust and government support affect the overall firm performance.

## 1 Introduction

The core of firm businesses depends on trading principles and is based on trust among both alliances involved in such phenomena [1]. Trust is the constitutes process among a firm's external and internal bodies, and stakeholders can accelerate firm performance [2]. Hence, as proceedings of digital transformation in all organizational fields to perform business activities, technological interference, specifically blockchain technology, plays a condemnatory role in improving firm performance [3]. Blockchain technology is considered a revolutionary, disseminated, and advanced transmissive technology that helps firms sustain coherence, confidentiality, and accessibility of all business proceedings and data [4]. Therefore, blockchain technology is an open and apportion ledger that can benefit organizations by recording data and negotiations approved by a cryptographic value among supply chain networks [5]. It is a contemporary web-based technology that can help firms to superior perceptibility among supply chain partners [6]. It accumulates transactional data in the form of blocks that are allocated among supply chain members. Hence, such blocks are combined cooperatively in a chronological sequence that creates a chain. Various units that are incriminated in any negotiation are entitled as a node, whereas the negotiation authentication is performed by cryptography [7]. A consolidated journal is successively cataloged and distributed between supply chain members [8]. Any negligence in accounting causes trust-related controversy among supply chain partners. Trust plays a positive role among supply chain members by delivering substantial promptness, authenticity, and other related factors; hence, trust-centered detriments could be excluded among members [9]. According to research conducted by Ref. [10], about 33 % of supply chain proceedings could be established through blockchain technologies. The consequence of such technological adoption is considered better progressive for accountability and perseverance of factors related to trust between supply chain partners [11]. Hence, it plays a role in transactional advancement, subsequently advantageous for supply chain members [12].

The researcher [13] in their study claimed that effectiveness and receptiveness are considered two significant supply chain constituents. At the same time, the supply chain exploits various contemporary technologies to procure a competitive advantage relative to their competitors [14]. Various researchers in their studies state that one of the crucial features of blockchain is its international scope, inherent transparency, and mediator's trust [15,16]. Hence, such characteristics are not identically crucial for all governments but more significant for governments that are more susceptible to corruption and lack of trust than other countries that firms and businesses highly trust. This shows that government influence plays a role in using blockchain technology [17]. The motivation for conducting this study lies in the increasing significance of supply chain management and blockchain technology in the manufacturing sector of Pakistan. The integration of blockchain technology has emerged as a potential solution to enhance supply chain parameters and create a trusted environment within firms. Trust among supply chain partners is essential for collaboration, information sharing, and joint decision-making, which can significantly impact firm performance [18]. Therefore, this study aims to bridge these research gaps by examining the role of supply chain parameters approved by blockchain technology in determining firm performance in the manufacturing sector of Pakistan. It specifically focuses on the moderating role of government support, as governmental policies and initiatives can either facilitate or hinder the adoption and implementation of blockchain technology in supply chain operations.

Despite the growing adoption of blockchain technology in various industries, including supply chain management, there is a lack of empirical research investigating literature regarding the specific supply chain parameters that are approved by blockchain technology and their impact on firm performance. While previous studies [[19], [20], [21]] have highlighted the benefits of using blockchain in supply chain management, few have delved into the specific parameters that can enhance firm performance within the manufacturing sector of Pakistan. Similarly, trust and government support are critical elements in supply chain relationships and can be significantly affected by the implementation of blockchain technology. To be able to fill the gap suggested by Ref. [6] in their study, our research study would provide a foundation to examine the impact of blockchain technology on the supply chain's triple-A parameters, such as adaptability, alignment, and agility. The current research study aims to discover the influence of supply chain parameters, including supply chain alignment, agility, and adaptability approved by blockchain technology, on a firm's performance with the mediating role of trust and the moderating role of government support. The current research study would deliver an understanding of the influence of supply chain parameters approved by blockchain technology from the perspective of the triple-A model developed by Ref. [22]. Moreover, it provides in-depth knowledge about supply chain parameters and how they influence the firm's performance.

Our study focuses on how blockchain technology provides transparency, traceability, and security to supply chain operations by creating a decentralized ledger that records transactions and information. Using blockchain, firms can reduce costs, increase efficiency, and improve their overall performance. Moreover, in various other research studies, the researchers have been influenced to investigate the impact of blockchain technology integrated with supply chain parameters on trust [23,24]. No one is used as a mediator to connect supply chain parameters to the firm's performance. Therefore, our study covers all aspects of adopting blockchain technology in supply chain management, which requires trust between supply chain partners. Hence, this research study would play a role in exploring such relation as many researchers advocate [20,25,26]. Lastly, moderating part of government support among trust and firm performance provides insights for firms and policymakers on enhancing their supply chain operations using government support. Furthermore, the study explains three research questions: first, do SCM parameters link to firm performance using blockchain technology in Pakistan? Second, does trust mediates the relationships between supply chain parameters approved by blockchain technology and firm performance? Third, does government support moderate the relationships between trust and firm performance?

## 2 Hypothesis development and theoretical foundation

### 2.1 Supporting theories

The resource-based view (RBV) theory is deliberated as the most significant theoretical foundation to understand how different firms achieve and sustain competitive advantages in the market. According to the RBV theory, an organization combines many different resources, whereas diversification of such resources governs the transformation of a firm's effectiveness [27]. These include internal and external factors associated with the firm, such as human assets, IT, equipment, and knowledge resources, which are considered both tangible and intangible. Moreover [28], studies state that different organizational and technological activities reflect significant resources to achieve sustainable competitive advantage. Manufacturer resources include management abilities, organizational information, and IT integration skills. Such resources help a firm earn an extraordinary profit, leading to an economic advantage. The RBV theory provides insight into the association between the firm's internal competitive advantage and capabilities. It gives a theoretical foundation for comprehending supply chain parameters approved by blockchain technology, including supply chain alignment, agility, adaptability, and their role in firm performance. Blockchain technology is considered a firm's resource in the RBV context, which helps organizations to accomplish sustainable competitive advantage by using such resources to improve the performance of the firm's activities. In their studies, the researcher [29] argued that IT, such as blockchain technology, has become one of the effective resources of a firm when accompanied by other activities and resources that lead to achieving a competitive advantage towards firm performance.

Blockchain-based reliable and efficient certificateless signature for IIoT devices explores the use of blockchain technology for implementing reliable and efficient certificateless signature schemes in Industrial Internet of Things (IIoT) devices [30,31]. It may provide insights into the security aspects of blockchain technology and its potential applications in the context of supply chain parameters in the manufacturing sector. Our study focuses on the use of blockchain technology in the emerging concept of the metaverse link with past studies [[32], [33], [34]] as they discussed various aspects of blockchain, including trust, security, and decentralization, and their relevance to the metaverse. While the metaverse is not directly related to the manufacturing sector [35], but these studies can provide insights into the broader applications of blockchain technology and its impact on different industries. Similarly, Blockchain and PUF-based lightweight authentication protocols for wireless medical sensor networks also present a lightweight authentication protocol for wireless medical sensor networks (WMSN) using a combination of blockchain technology and physically unclonable functions (PUFs) [36]. While the specific application is related to healthcare and sensor networks, it can offer insights into integrating blockchain technology with other security mechanisms and its potential relevance to supply chain parameters and firm performance, as evidenced by previous studies [37,38] supported our arguments.

### 2.2 Supply chain parameters approved by blockchain technology and firm performance

#### 2.2.1 Supply chain alignment and firm performance

A supply chain refers to a network of suppliers and customers who perform their work collectively to deliver any precise service to end users [39]. Alignment is considered consistent among strategic objectives, metrics, and accomplishments [40]. Alignment is a fundamental predecessor that plays a significant role in the firm performance through various business and organizational disciplines, which include strategy, management information systems, organizational behavior, and manufacturing strategy [41]. Hence, supply chain alignment helps firms to achieve organizational objectives, structures, and practices inside and among diverse tasks and participants of a supply chain which consequently primes towards improving the firm performance. Several research studies have proved the significance of supply chain in their findings, whereas the researcher [22] demonstrates that supply chain alignment is considered one of three intended business constraints apart from the other two supply chain parameters, such as agility and agility adaptability. Functional silos and competing marketing, trading, and manufacturing objectives lack supply chain alignment [42]. Moreover, the author [6] in their study proposed that supply chain alignment is the method by which different supply chain partners incorporate with one another to develop the performance of firms. Hence, there should be alignment among supply chain policies and internal and external supply chain members. Various other studies also state that supply chain alignment enhances the value of a customer and competitive advantage, ultimately resulting in improved firm performance [43].

In their study, the researcher [44] stated that blockchain could integrate all member partners' supply chain processes. All supply chain partners can view the internal processes of all supply chain parties. In such a way, blockchain also helps accelerate the promptness of the performance of business processes with higher precision and dependability [45]. It facilitates firms to share proceedings and records with all supply chain parties, enabling them to abolish trust-related issues among associated parties aligned supply chains towards common business objectives [6]. Out of many advantages of supply chain alignment in blockchain technology, some of them include the possibility to align all the suppliers' tracks, their identities, and acceptability in the market, and to negotiate using smart contracts automatically, the best prices in real-time while considering the seller's position [46]. Secondly, blockchain technology will lead firms to enhance perceptibility, supply chain advancement, and better demand estimating. Supply chain alignment increases the transparency of all associated parties, making it feasible to counter in real-time any uncertain situation [47]. In this way, it decreases the error and fraud rate and improves inventory management [48]. Past research [20] still lacks an understanding of various types of supply chain alignment accomplished with market behavior. Hence, the studies of [39] help to answer the following gap by acknowledging two different sorts of shareholder alignment (SA) and second one customer alignment (CA). It is reasonable to investigate the SA and CA since there must be an alignment among supply chain strategies and members of different supply chains, both internally and externally. SA involves the functional approaches and business practices used to keep them constant with the expectation of shareholders and business strategy, including increased revenue, efficient working capital, and decreased operational cost [49]. Hence, it contributes to effective firm performance by aligning all internal supply chain processes with the strategic organizational objective. CA is the process in which business strategies and supply chain strategies are aligned to enhance the customer, improving firm performance [50]. Consequently, we hypothesized, based on the preceding discussion, that;

H1a Supply chain alignment is positively linked to firm performance using blockchain technology in Pakistan.

#### 2.2.2 Supply chain agility and firm performance

Agility is an inclusive, multifaceted concept that includes various other concepts such as customer, operational, organizational, enterprise, and supply chain agility [51]. Iacocca Institute proposed the term agility from the business perspective by proposing that a firm develops in an environment where it could transform rapidly and impulsively by performing its business activities in an agile way. In contrast, some other researchers consider it a privilege because it could provide an advantage to its customers, promptly cope against market changes, esteem human understanding and abilities, and develop cybernetic businesses [52]. Supply chain agility consists of the internal and external competencies of a firm in association with the firm's strategic customers and suppliers to encounter market fluctuations and perspectives as well as real interferences responsively [53]. Hence, supply chain agility could be accomplished through collaborations of diverse types of tractability from all supply chain members [54]. It depends on various diverse characteristics which influence the firm performance, which includes the speed of delivery, consolidated and cooperative planning, data precision, vendor administration inventory placement, quick-response approach, a decline in lead time, cost depreciation, IT integration, including blockchain technologies, and organizational flexibility [51]. Furthermore, a study [53] suggested that the supply chain contains a succession of interrelated events, including designing, producing, and distributing particular products or services between different supply chain parties. In this way, firms must cooperate with channel members to execute these allied activities proficiently and cooperatively govern the market volatility to gain a competitive advantage. It showed that supply chain agility is entirely associated with customer receptiveness in any tentative marketplace [55].

Moreover, confirming the firm's effectiveness is crucial because supply chain agility empowers effective and proficient reactions against operational fluctuations such as acquisition, manufacturing, distribution, and advertisement [56]. It helps organizations and businesses gain customer receptiveness and excel in market fluctuations using IT integration, including blockchain technology [57,58]. The IT integration helps supply chain partners to develop discernibility and empowers firms to recognize market changes, eventually decreasing the cost of demand insecurity. Likewise, supply chain agility facilitates firms to synchronize through different members through a common vision about development and business practices, decreasing prospective conflicts and adaptable behaviors in the supply chain [59]. Subsequently, it influences the firm to organize resources with other members that develop the adeptness of products and service provision. Therefore, it improves the daily functioning of firms, decreases costs, and increases effectiveness [60]. A past study [61] stated that supply chain agility denotes the supply chain's ability to sustain planning constraints in unnatural conditions. The supply chain's agile capability highlights the likelihood and prevention, and it should attain the most suitable match among substantial resources of the firm and the application of such resources by advanced behaviors [62]. Hence, the supply chain agility approved by blockchain technology is considered a crucial factor towards effective firm performance. Based on such discussion, we hypothesized that;

H1b Supply chain agility is positively linked to firm performance using blockchain technology in Pakistan.

#### 2.2.3 Supply chain adaptability and firm performance

Supply chain adaptability is a firm's ability to detect long-term, critical fluctuations in the market environment. Supply chain adaptability referred socio-political changes, economic changes, demographical changes, and revolutionary technological progress that react against these fluctuations by adaptably regulating the conformation of the supply chain [63]. These include emerging new supply centers, repositioning production accommodations, and outsourcing [64]. Some research studies [65] claimed that innovativeness is an important aspect of supply chain adaptability because innovation is a way to change an organization against fluctuations in the organizational environment. Supply chain adaptability highlights innovativeness relative to the firm capability to bring various innovative products, procedures, and technologies into the market. A researcher [22] argued that supply chain adaptability substantially affects the firm's market and financial performance from the supply chain performance. At the same time, few studies state that supply chain adaptability results in superior firm performance. Various research studies [66,67] claimed that supply chain adaptability is conceived as a high-order dynamic ability that influences the firm performance and enables the reconstruction of organizational resources and maximizing environmental changes, strengths, and weaknesses of the firm. The dynamic competencies enable a firm to imitate resources that consequently bring sustainable competitive advantage. Hence, supply chain adaptability results in superior occupational performance [68].

Supply chain adaptability significantly benefits cost and firm operational performance [22]. Past researchers have considered supply chain adaptation as a vigorous ability that delivers a framework for advancing and improving a firm's product or service novelty competencies and minimizes the associated product risk [69]. The firms actively involved in supply chain adaptation are considered the first mover and hence gain a competitive advantage against other firms. Many past studies have emphasized the role of blockchain technology in the supply chain mixture as well as the performance of the firm [70,71]. Blockchain technology helps the firm cooperate with other chain members and assist in sharing data that ultimately sense changes in the marketplace and make rapid changes in the firm, thus effectively influencing the firm performance [2]. Moreover, a research study [44] argued that blockchain technology has some intrinsic properties to rapidly incorporate all supply chain processes, which influence firm performance. The adaptability of blockchain technology is proved significant for creating a more precise prediction of demand, inventory management, and reserves in case of any alterations in the market environment [72]. In such a way, blockchain technology authorizes the firms to respond to fluctuations in design and suppliers promptly; along with that, the quality documentation could also be systematized and distributed with all supply chain parties, positively influencing the firm decision-making and consequently improving the firm performance [73]. Such technological improvement helps supply chain members to share and exploit all documentation related to designing; hence some firms have initiated the integration of manufacturing with blockchain technology, GPS could be integrated with blockchain and logistics could also be governed by such technology [74]. Based on such discussion, we hypothesized that:

H1c Supply chain adaptability is positively linked to firm performance using blockchain technology in Pakistan.

### 2.3 The mediating role of trust

One of the primary hindrances to individuals is not purchasing online because they feel insecure about providing personal data to digital payment services [75]. Trust in digital purchasing that demand information from customers, as well as customer comfort and satisfaction. For the relationship to be successful, customers should be educated regarding the company's data collection practices and policies [25]. On the other side, trust is the only factor that connects the client to share personal information for an organization to build customer relationships. Trust among firms produces an environment where all companies work to exceed minimal necessities [76]. Previous studies [77] agreed that trust is a complex issue involving perceptions of the system players' honesty, reliability, competency, and dependability to be trusted. A prior study has examined the significance of trust at the interpersonal and inter-organizational stages [78]. The trust level between individuals in different organizations is known as interpersonal trust. Similarly, the level of trust between individuals within one organization to another is known as inter-organizational trust. In this study, we describe companies as flexible agents interacting with partners and changing over time inside a supply chain network. Remember that the downstream agent chooses one or extra upper-stage mediators based on their trust value. As a result, this essay focuses on interpersonal trust, which results from repeated prior interactions between two people that help them get to know and develop a sense of commitment to one another [79]. Blockchain potentially reduces communication or data transfer errors by providing access to all participants in a given supply chain, affecting organizations' performance [80].

The current study explains trust is essential in supply chain parameters, especially in blockchain technology cases [81]. Supply chain alignment and individual trust lead to coordinating all activities resulting in supply chain partners and stakeholders having a shared objective and working toward common goals to achieve the firm performance [82]. Supply chain agility with trust denotes the supply chain's constantly changing needs; a firm should be able to quickly change its scheme, particularly regarding delivery, inventory control, and purchasing [83]. When supply chain adaptability is covered with users' trust, it designs to adapt to disruptions, structural changes, and changing consumer trends behavior. The ability to modify each supply network must account for such modifications to increase the organization's performance [84]. Supply chain trust serves as an unofficial bridge between businesses. Even if it may harm them personally, the supply chain as a whole will benefit when all its participants trust one another [85]. A competitive advantage could be gained by having crucial skills like alignment, adaptability, and agility. A resource must be valuable, uncommon, unique, and non-replaceable to contribute towards the growth of sustainable firm performance [86]. A blockchain platform introduces standard, trusted data, minimizing duplication and improving supply chain visibility [15]. Blockchain technology encourages the development of trust between parties that impact the company's performance [87]. That is why the current study covers the supply chain parameters approved by blockchain technology with people's trust to improve organizations' performance. Through the above discussion, it hypothesized;

H2 Trust positively mediates the relationships between supply chain parameters approved by blockchain technology and firm performance.

### 2.4 The moderating role of government support

Government support refers to a legislative context created to control how service companies fulfill their legal commitments and protect consumers from fraud and infractions [88]. Government instructions and restrictions can influence people's decisions to buy supplies and goods in a peaceful environment [89]. The consumer's behavioral goals to pursue blockchain technology are supported by government support in a past study where researchers were awarded that utilizing technology could be beneficial and increase productivity. However, it is still required to allow and regulate its adaption through trust [90]. Government support moderates the relationship between trust and firm performance, although consumers do not fully recognize the convolution and suggestion of new technology in supply chain parameters. However, due to legal security, they will recognize and implement it [91]. Government and public sector organizations are utilizing supply chain management to its maximum capacity to provide value to the public more effectively and efficiently and increase the performance of organizations [92]. The trust factor ensures that operations and supply chain management techniques are set up for success, enabling them to thrive today and in the future and remain resilient in times of change [93]. Government is responsible for assisting the people to trust crucial components of supply chains and the manufacturing workforce. These initiatives will give people higher-quality jobs and make the labor market more responsive to expanding businesses [94]. Supply chain governance is comprehensive and involves establishing, growing, and upholding connections between blockchain technology and firm performance. Trust in the supply chain organizes the distribution of resources, financial, material, and individual processes, and frameworks for making important decisions [95].

Government offers different pieces of training and short courses for individuals to cover supply chain techniques [96]. Government support bridges people's trust in supply chain goods and their organization's performance. People feel more secure when government protects their common goods [83]. Through supply chain alignment, agility, and adaptability, people work towards the same goals and behave in a coordinated manner [97]. Clients trust suppliers to transport products on time and at the consensus cost [98]. Additionally, buyers would show their gratitude to their suppliers by making every effort to pay on time per the terms set forth [99]. A high level of trust benefits businesses by improving financial performance and increasing employee engagement [100]. A previous study [101] explained that government support rules and regulations are applied directly to companies, and other aspects like competitive market factors between corporations and citizens are also considered. Another prior study described the moderating role of government support, which gave the confidence to trust products and raise their organizations performance to strengthen our study arguments [102]. Our study emphases on moderating role of government support among trust and firm performance in perspective of supply chain parameters. Individuals who have trusted the supply chain goods along government support, if they are assured government all products provided with proper rules and regulations and on time that ultimately increase our firm performance. Through above discussion it hypothesized;

H3 Government support positively moderates the relationships between trust and firm performance.

### 2.5 Theoretical framework

**Fig. 1:**
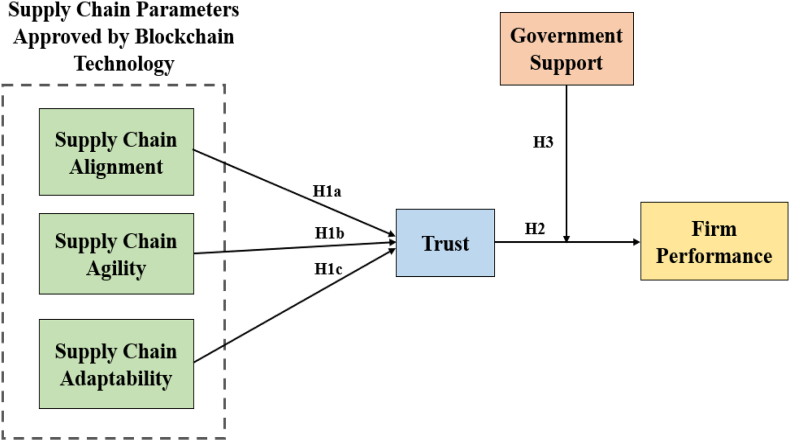
Theoretical framework.

Considering the theoretical context and literature review for developing the hypothesis followed by RBV theory, the present research study has proposed a novel framework for unveiling the role of supply chain parameters approved by blockchain technology towards firm performance. In addition, the mediating role of trust and moderating role of government support towards firm performance and supply chain parameters are approved by blockchain technology. Fig. 1 represents a research framework in-depth, as in the literature review introduced above, to clarify the model shown below.

## 3 Material and methods

This study concentrates on the supply chain parameters (alignment, agility, and adaptability) approved by blockchain technology and firm performance through trust and government support in Pakistan. The target market consists of supply chain professionals working in manufacturing industries listed on Pakistan Stock Exchange. This study concentrated on Pakistan because they have faced an economic crisis and need improvement in the industrial zone. The target sector was mainly the manufacturing sector, including top textiles industries from Pakistan. The chosen textiles industries because it plays a significant role in Pakistan's economy, contributing to GDP growth, employment generation, and export earnings [45]. This study might be helpful for manufacturing sectors to focus on their supply chain goods through blockchain adoption because this sector contributes to the country's business and GDP contribution. Pakistan's manufacturing sectors contribute 8.5 % of the country's annual GDP [103,104]. In the developing world, the manufacturing sectors have a large potential to boost economic growth in a country like Pakistan, so we chose the textile sector to adopt blockchain. The selection of manufacturing firms listed on the Pakistan Stock Exchange suggests that the researchers are interested in studying publicly traded companies subject to regulatory oversight and reporting requirements. PLS-SEM is a statistical technique commonly used in social science research to analyze relationships between latent variables. The SmartPLS 4.0 techniques are used based on previous studies [105,106], which provided an appropriate setting for the current study. Using Harman's single-factor check, the study investigates typical technique bias. There is no issue of common bias in the research if the sum of variation for a single element is >50 %, but there is a problem of bias in the data if it is <50 % [107]. However, our investigation discovered (15.8 %), indicating no problem with bias in the research data compared to the recommendations of a single-factor check.

### 3.1 Data collection procedure

In our study, we considered the complexity of our model, the number of latent variables, and the available resources to determine an appropriate sample size. PLS-SEM is known for its flexibility and ability to handle small sample sizes, non-normal and non-linear data, and models with many variables or complex relationships [108]. The population size is enormous, and it isn't easy to approach each participant; a convenient sampling technique was utilized in this study as an effective medium [109]. Convenience sampling participants are chosen based on their availability and willingness to participate. Online surveys frequently employ convenience sampling since contacting respondents through email, social media, or other online channels is simple. The questionnaires were distributed and reconciled during the survey administration through emails and e-messages. We collected data from the managers of several production organizations using online and offline structured questionnaires. They were requested to respond and share their opinions on the effectiveness of the elements that had been investigated in their organization using a five-point Likert scale with a range of 1 (“strongly disagree”) to 5 (“strongly agree”). In the survey, junior, middle, and top executives from manufacturing (textile) firms were asked to share their thoughts on the relevance of adopting supply chain parameters (alignment, agility, and adaptability) approved by blockchain technology and firm performance through trust and government support helping them become more competitive on the operational and environmental frontiers. For this purpose, the process of gathering data was started in the first quarter of 2023.552 questionnaires were initially distributed to participants, and we received 465 responses, of which 87 were missing. Hence, this represents a response rate of 84.2 %. The sample size supported the recommendation of research [110] for a minimum of 150 sample sizes for the SmartPLS 4.0 technique; hence it was sufficient to draw a conclusion. PLS-SEM is a high-level modeling strategy that investigates the relationship between the PLS algorithm and the inner model using t-values. Out of 465 respondents, 67 % are males, and 33 % are females. The educational details of respondents comprised 14 % having intermediate degrees, 40 % having bachelor's degrees, 35 % having master's degrees, and 11 % having doctoral degrees. The sample's composition with a designation included 14 % of participants working in higher executives in firms. 33 % were working as middle executives, and 56 % of participants were employees of firms working as junior executives. Participants' Professional experience contained 31 % of respondents with 0–5 years of experience; 54 % of participants with 6–15 years of experience are employed, and 15 % of respondents with 16–25 years of experience are employed. Further details are described in Table 1.

**Table 1:** Demographic details.

Demographics	Distribution	n = 465
Gender	Male	311 (67 %)
	Female	154 (33 %)
Age	18–25 years	171 (37 %)
	26–35 years	225 (48 %)
	More than 35 years	69 (15 %)
Qualification	Intermediate	64 (14 %)
	Bachelor	186 (40 %)
	Masters	163 (35 %)
	PhD	52 (11 %)
Job Level	Higher executives	96 (21 %)
	Middle executives	221 (48 %)
	Junior executives	148 (31 %)
Experience	5 or less years	143 (31 %)
	6–15 years	251 (54 %)
	16–25 years	71 (15 %)
Industry (Textile Companies)	Gul Ahmed Textile Mills Limited	71 (15 %)
	Fateh Textile Mills	80 (17 %)
	Chenab Limited	83 (18 %)
	Nishat Mills Limited	69 (15 %)
	The Crescent Textile Mills Limited	85 (18 %)
	Kohinoor Mills Limited	77 (17 %)

### 3.2 Construct measurements

All variables considered in this investigation were mostly adapted from earlier research studies to fulfill the requirements of the literature on information sharing. The supply chain's parameters effectively solve problems like increasing demand and supply in uncertainty and connect with several external stakeholders and coordinate their requirements and interests [111]. First, supply chain parameters were established and developed from the literature [81]. Supply chain parameters were divided into three sub-dimensions: alignment, agility, and adaptability. Six items of supply chain alignment were accepted from Ref. [19]. Five items of supply chain agility were taken from Ref. [81]. Five items of supply chain adaptability were adopted from Ref. [20]. Second, the integrity of supply chain parameters is ensured by mutual trust, and people are ready to buy goods with respect and do payments with satisfaction [112]. Trust five items were assessed by Ref. [75]. Third, government support covers the legal frameworks put in place for the recognized bodies to supervise, ensure that technology users carry out their duties, and prevent violations [95]. Government supports four items were taken from Ref. [91]. Last, firm performance refers to activities that include available products, distributions completed on time, and the supply has all inventory and bulk to provide performance on time [113]. Firm performance six items were assessed by Ref. [26]. In Appendix A, the detail of the questionnaire is provided. The PLS-SEM analysis technique was used in this study to examine both observed and unobserved variables and indicators.

## 4 Data analysis and findings

The current study uses partial least square structural equation modeling to measure the proposed model of the supply chain through Smart PLS 4.0 statistical analysis software. Two main components to partial least squares structural equation modeling (PLS-SEM) exist. First, the measurement model is commonly known as the outer model. The structural model, referred to as an inner model, is the second one [114]. A causal forecasting system evaluates statistical models to describe connecting relationships. This technique has many advantages. PLS-SEM effectively handles small sample sizes, numerous variables, and related questions by determining the independent regression analysis for measurement and structural models [115].

### 4.1 Measurement model

The validity and reliability tests are first examined to determine the acceptability of a measuring tool. Validity denotes the representation of variables of a specific concept, whereas reliability determines the consistency in outcomes assessed using the instrument [116]. We calculated the reliability and validity of successfully implementing the constructs with various methods. The results shown in Table 2 indicate the outer model's regular factors loadings were over the minimum threshold of 0.70, as Cronbach's alpha and composite reliability are used to evaluate the internal consistency of all the variables. It indicates how closely constructs are connected to each other [117]. The threshold value is 0.70, and all items meet the range in Table 2. The AVE measured the convergent validity of each construct. Its minimal cutoff value was 0.50 or more [118]. The AVE of all items meets the range in Table 2. We looked into the probability of multicollinearity by analyzing the acceptance and variance inflation factor (VIF). The low threshold number implies that the measurements are accurate. The threshold value of VIF needs to be less than 5 [119]. Table 2 of VIF demonstrates that the dataset's multicollinearity was relevant. Furthermore, all constructs' Cronbach's alpha, composite reliability, and AVE exceeded the threshold values, demonstrating our obtained results' accuracy and convergence.

**Table 2:** Factor analysis, validity, reliability and collinearity statistics.

Latent constructs	Items	Factor Loadings	α	CR	AVE	VIF
Supply chain alignment (SCAL)			0.939	0.952	0.766	
	SCAL1	0.882				2.408
	SCAL2	0.863				2.914
	SCAL3	0.874				3.044
	SCAL4	0.928				2.863
	SCAL5	0.864				2.833
	SCAL6	0.838				4.113
Supply chain agility (SCAG)			0.938	0.953	0.803	
	SCAG1	0.876				2.943
	SCAG2	0.925				4.330
	SCAG3	0.940				3.421
	SCAG4	0.922				2.758
	SCAG5	0.812				2.145
Supply chain adaptability (SCAD)			0.907	0.931	0.731	
	SCAD1	0.718				1.789
	SCAD2	0.875				2.863
	SCAD3	0.920				4.813
	SCAD4	0.879				2.843
	SCAD5	0.870				3.917
Firm performance (FP)			0.896	0.921	0.658	
	FP1	0.838				2.721
	FP2	0.832				2.565
	FP3	0.800				2.075
	FP4	0.762				1.935
	FP5	0.839				3.363
	FP6	0.792				2.681
Trust (T)			0.939	0.954	0.806	
	T1	0.842				2.339
	T2	0.847				2.547
	T3	0.924				4.867
	T4	0.942				3.670
	T5	0.929				4.156
Government support (GS)			0.969	0.977	0.916	
	GS1	0.953				1.211
	GS2	0.921				2.721
	GS3	0.980				3.612
	GS4	0.972				2.152

**Note:** FL = Factor Loadings, VIF = Variance inflation factor, CR = Composite reliability, AVE = Average variance extracted, α = Cronbach's alpha.

### 4.2 Discriminant validity

Discriminant validity was examined to determine whether the utilized constructs differed from one another [108]. We used a variety of approaches to estimate the discriminant validity. We examined the connection between the extracted average variance (AVE) and the share variance of constructs. Table 3 demonstrates that for all constructs, the root of the AVE was higher than the correlational values. Additionally, the Heterotrait-Monotrait (HTMT) ratio, which is determined by a standard way of DV analysis, was calculated [115]. The threshold value of HTMT should not exceed 1 [120].

**Table 3:** Discriminant validity.

Constructs	FP	GS	SCAD	SCAG	SCAL	T
FP	**0.811**					
GS	0.743	**0.957**				
SCAD	0.855	0.823	**0.855**			
SCAG	0.157	0.149	0.191	**0.896**		
SCAL	0.418	0.311	0.373	0.146	**0.875**	
T	0.481	0.389	0.443	−0.053	0.406	**0.898**

Note: FP = Firm performance, SCAD = Supply chain adaptability, GS = Government support, SCAG = Supply chain agility, T = Trust, SCAL = Supply chain alignment.

### 4.3 Structure model

**Fig. 2:**
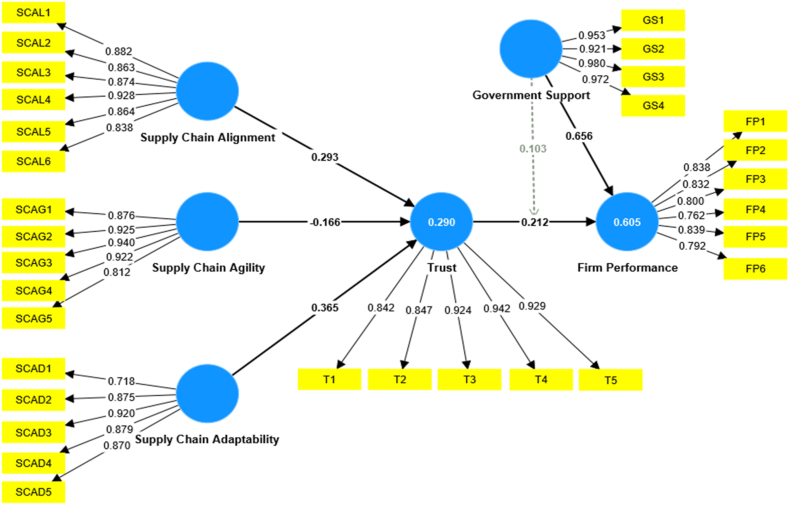
Estimation model (Partial least squares (PLS-SEM) algorithm).

Structural equation modeling (SEM) was used to evaluate the hypotheses and respond to the relevant research problems for the structural model. The significance of the hypotheses was investigated using the bootstrapping method [108]. Firstly, it was evaluated for path coefficient values and R^2^ to determine the predictive value of the study model assessment. Correspondingly, R^2^ analysis determines the coefficient of determination using the dependent variable's total variance and variation ratio on independent constructs [115]. The R^2^ value of the current study model shows in Fig. 2 as firm performance = 0.60 % and trust = 0.29 %. A past researcher [121] provided a research tool that was utilized to calculate the goodness of fit (GOF). The values of GOF indicate that the model is overall a good fit. For representation NFI = 0.811, SRMR = 0.068 and Chi-Square = 2678.235.

### 4.4 Hypothesis testing

We applied structural equation modeling (SEM) analysis using the PLS bootstrapping method to test our study hypotheses. The results were evaluated using t-statistics, standardized path (Beta coefficient) values, and p-statistics [114]. These results reveal that the particular variable has a favorable or unfavorable impact on other variables. It demonstrated the strong direct and indirect effects that some independent constructs on the dependent constructs have on one another [116]. The constructs in the structural model are supply chain adaptability, government support, supply chain agility, trust, supply chain alignment, and firm performance. The relevance of the variables was calculated using the p-value criterion (p < 0.05). Table 4 offers an overview of discoveries. The hypothesis testing outcomes showed that supply chain alignment positively impacts trust with values (β = 0.293, t = 6.133, and p = 0.000), respectively. Supply chain agility positively impacts trust with values (β = −0.166, t = 2.919, and p = 0.004). Supply chain adaptability positively impacts trust with values (β = 0.365, t = 8.533, and p = 0.000), respectively. Consequently, the study outcomes have supported H1a, H1b, and H1c. Trust positively impacts firm performance with values (β = 0.212, t = 5.927, and p = 0.000), respectively. Government support positively impacts firm performance with values (β = 0.656, t = 24.483, and p = 0.000), respectively. This study determines the mediating role of trust between supply chain parameters and firm performance. Furthermore, trust positively mediated the relationship between supply chain alignment and firm performance (β = 0.062, t = 4.189, and p = 0.001), respectively. Trust positively mediated the relationship between supply chain agility and firm performance (β = −0.035, t = 2.830, and p = 0.005), respectively. Trust positively mediated the relationship between supply chain adaptability and firm performance (β = 0.078, t = 4.206, and p = 0.002), respectively. Therefore, the study results have supported H2. Additionally, this study calculates the moderating effect of government support which positively moderated the relationship between trust and firm performance (β = 0.103, t = 2.969, and p = 0.000). Hence, the findings confirmed the validity of H3. All hypotheses of this study are supported.

**Table 4:** Hypothesis testing.

Hypothesis	β-values	Mean	STDEV	t-values	p-values	Results
Direct effect						
GS - > FP	0.656	0.655	0.027	24.483	0.000	Accepted
SCAD - > T	0.365	0.363	0.043	8.533	0.000	Accepted
SCAG - > T	−0.166	−0.160	0.057	2.919	0.004	Accepted
SCAL - > T	0.293	0.296	0.048	6.133	0.000	Accepted
T - > FP	0.212	0.214	0.036	5.927	0.000	Accepted
**Mediation effect**						
SCAD - > T - > FP	0.078	0.079	0.018	4.206	0.002	Accepted
SCAG - > T - > FP	−0.035	−0.034	0.012	2.830	0.005	Accepted
SCAL - > T- > FP	0.062	0.063	0.015	4.189	0.001	Accepted
**Moderating effect of GS on FP**						
T*GS - > FP	0.103	0.106	0.035	2.969	0.003	Accepted

Note: FP = Firm performance, SCAD = Supply chain adaptability, GS = Government support, SCAG = Supply chain agility, T = Trust, SCAL = Supply chain alignment.

**Fig. 3:**
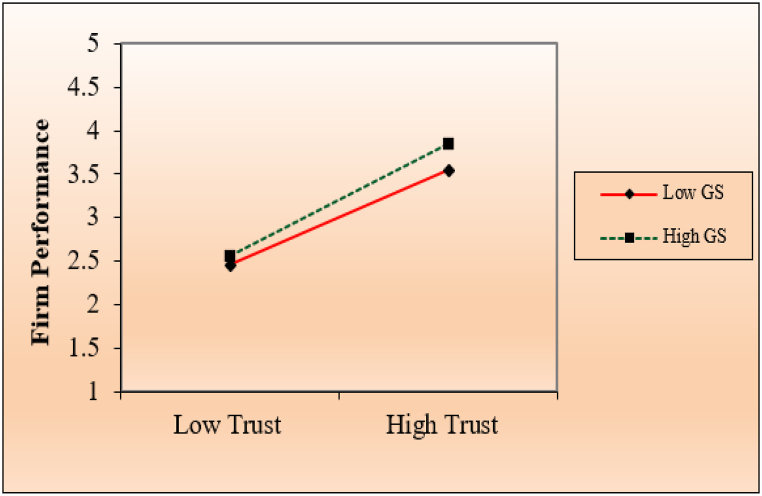
Moderating graph of government support.

Furthermore, we provided two distinct association graphs to interpret the moderation outcomes. The government support moderation effect among trust, and firm performance was explained in Fig. 3. The graph demonstrates the relationship between trust and firm performance under high government support. The distinguished point on the moderation graph is a firm performance at a high and low level of government support. The association among trust and firm performance are high when government support is high in Fig. 3, which means trust and government support are consistent.

## 5 Discussion

The main goal of this study is to inspect the impact of supply chain parameters such as supply chain agility, adaptability, and alignment on firm performance with the mediating role of trust in the Pakistan context. It further inspects the contribution of government support as a moderating variable among trust and firm performance. To analyze the following relationship, researchers formulated three hypotheses, and data was collected by combining online and physical usage survey questionnaires completed by a sample of 465 employees of industrial firms listed in the PSE market. The research study has three significant research findings. Firstly, the research finding states that supply chain parameters, including agility, adaptability, and alignment, significantly positively correlated to firm performance using blockchain technology in Pakistan. Past research studies also claimed that the firms that use blockchain-enabled supply chain parameters to perform their processes eventually lead towards positive firm performance [2,9,122]. The research study of [6] supports our study by proving that blockchain-empowered supply chain parameters such as alignment, adaptability, and agility positively influence economic advantage, improving firm performance. It depicts those managers working in firms who have a notion that using blockchain technology would assist their business to become agile by minimizing the production lead times, accelerating the development speed of new products, aggregating delivery abilities, and enhancing customer satisfaction. Correspondingly, in the perspective of alignment, they perceive that by adopting blockchain technology, managers could incorporate the firm's sourcing, delivery, and provision process and complete the firm's performance [55]. Moreover, regarding adaptability, they assume blockchain technology would enable managers to control product mix according to market demand. There is a positive affiliation among blockchain improved supply chain adaptability, agility alignment, and firm performance [22,51].

Secondly, this study scrutinizes the mediating role of trust among supply chain parameters approved by blockchain technology and firm performance. These results proved that trust significantly mediates the relationship between supply chain parameters approved by blockchain technology and firm performance, supported by many previous studies [6,20,123]. A previous study [124] clarified trust played a crucial role in enlightening firm performance among supply chain members. If supply chain participants lack trust in one another, then it creates a major obstacle to collaboration among different departments of firms in the supply chain. Hence, firms should put some effort into developing trust among channel participants in the supply chain, which would eventually increase the overall performance of a firm. Besides this, studies by Ref. [75] also state that trust is an integral factor in the supply chain as it helps in time reduction, increased flexibility, and enhanced customer satisfaction. At the same time, few studies [125] stated that trust is a more efficient and less costly resource to protect specialized market investments, particularly regarding the supply chain. Therefore, creating trust among supply chain participants would also improve firm performance, as supported by this study. Hence, many researchers have examined it in their studies. Still, we have specifically investigated the mediating role of trust among supply chain parameters and firm performance, which positively mediates their relationship, making our study distinctive from other studies [77,126].

Thirdly, this study considers the moderating relationship between government support among trust and firm performance. The analysis results show that government support positively moderates the relationship between trust and firm performance. Various other studies [127] also support our study by examining that the governments of different nations have explored the positive significance of integrating blockchain technology in their public and private firms, transforming organizations towards digitalization and eventually impacting firm performance. Past researchers perceived that integrating supply chain-based blockchain has prospective welfare for the government, which includes enhanced data quality, data reliability, clearness, lower chances of deception and errors, lower corruption rate, and increased trust [90,93,94]—considering all the benefits the government support firms to use supply chain parameters approved by blockchain that enhance the performance of firms [17] whereas [122], in their study state that government support for using blockchain technology in their supply chain is primarily due to the benefits associated with the technology. That involves the transparency and trust factor, which increased blockchain integration. Many other researchers also argue that blockchain technologies in both the public and private sector increase firm efficiency and performance [91,99]. Hence, our research study also proved that government support positively moderates the relationship between trust and firm performance. The findings of this study shed light on the importance of blockchain-enabled supply chain parameters in improving firm performance in the manufacturing sector of Pakistan. By leveraging all supply chain parameters, manufacturers can enhance operational efficiency, reduce costs, and improve customer satisfaction.

### 5.1 Conclusion and policy recommendations

Trust and government support are considered crucial features for a combination of supply chain parameters approved by blockchain technology to improve firm performance. Still, there are scarce studies that simultaneously discourse the following relationship. The current research study aims to explore the role of supply chain parameters approved by blockchain technology toward firm performance with the mediating role of trust. Moreover, the moderating role of government support is also investigated, contributing to the body of literature. The research study has a three-fold contribution; First, supply chain parameters approved by blockchain technology, such as supply chain alignment, agility, and adaptability, have a major positive relationship with firm performance. Second, trust positively mediates the relationship between supply chain parameters approved by blockchain technology and firm performance. Lastly, government support positively moderates the relationship among trust and firm performance. Firm performance is the key concern for any firm; hence the assimilation of supply chain-enabled blockchain technology helps organization managers cope with various external and internal problems they could resolve using such technology.

### 5.2 Theoretical and practical implications

The study has several theoretical for research scholars, managers, and organizational bodies. Some important implications of theory and practice are deliberated to support the country's industry. The theoretical implications include: It provides a comprehensive overview of blockchain technology-enabled supply chain parameters, considered a contemporary technological aspect authorized to revolutionize supply chain channels. It comprises in-depth practical knowledge of blockchain technology and enduring industrial endeavors to integrate such technology to perform supply chain activities that eventually improve the firm performance. Secondly, the research would add value to supply chain administration theory by engaging the embryonic conception of supply chain parameters approved by blockchain technology. It proposed the framework with some other set of propositions that future researchers can study to study for empirical substantiation and supplementary extension. Therefore, supply chain parameters approved by blockchain technology will develop in the future. Lastly, this study highlights the moderating effect of government support on the relationship between individual trust and firm performance. These findings shed light on the significance of government policies and initiatives in promoting the adoption of blockchain technology and supporting firms to leverage its benefits. It allows policymakers to develop tailored strategies to encourage blockchain implementation in manufacturing and stimulate overall economic growth. Hence, we suggest researchers investigate the proposed framework to test other real-world problems.

Our current study offers some practical implications. First, the research study assists managers and organizational bodies with the context of blockchain technology in their supply chain members. Hence, supply chain parties, particularly managers, can voluntarily exploit research findings in their respective industrial contexts. Particularly, our study highlights the requirement of different types of blockchain technology-enabled supply chain efforts in a different industrial context. Such information assists supply chain managers in forecasting how to locate a firm's blockchain efforts per their industry requirements. Secondly, it provides grounds to supply chain managers regarding the abilities and performance products a firm could achieve by integrating blockchain technology at internal and external organizational levels. Thirdly, supply chain managers can effortlessly transform the theoretical situation associated with the resource-based view theory and how managers could best use this theory to make better perquisites for building managerial decisions regarding resources and abilities from the perception of the firm and supply chain. Consequently, supply chain managers could empirically exploit the proposed resource-based view integrated model as a managerial instrument to control firms on how they could advance and establish blockchain-approved supply chain capabilities among their supply chain parties. Hence, we antedate that the following theoretical and analytical understandings that this research study could contribute would prove constructive for both researchers and specialists to recognize the future advancements of blockchain technology applications in the supply chain context. Fourthly, this study provides sector-specific insights for manufacturers in Pakistan. Firms can draw from the research findings to tailor their supply chain strategies, considering the unique characteristics and challenges in the country's manufacturing sector.

People will invest globally in Pakistan's manufacturing sector through government support and facilitations. They will get the benefits of blockchain technology effectively; manufacturing firms may need to invest in training and capacity-building initiatives. Managers of organizations arrange training and develop the necessary skills and expertise to manage and utilize blockchain systems that will be crucial for successful implementation and maximizing its potential in supply chain management. This research study in the manufacturing sector of Pakistan and other countries can contribute to the broader global supply chain industry. Understanding the role of supply chain parameters approved by blockchain technology and the moderating effect of government support can extrapolate insights from this research to other regions and industries. This can inform best practices, guide future investments, and facilitate knowledge sharing among international supply chain practitioners. The study highlights the dynamic nature of the supply chain environment and the need for continuous learning and adaptation. Therefore, managers should explore and adopt blockchain solutions in their supply chain processes. Managers should prioritize building strong relationships with supply chain partners and utilize blockchain-enabled solutions to establish stakeholder trust. Collaborating with the government can provide access to resources, facilitate technology adoption, and create an enabling environment for supply chain improvements. They should invest in employee training and development programs to enhance their skills and knowledge.

### 5.3 Limitations and future directions

There are a few restrictions to this study. Firstly, the data comprised only manufacturing, especially textile companies registered on the Pakistan Stock Exchange. Hence, future researchers are suggested to develop the conceptual framework by testing it in different industries such as FBT (food, beverages, and tobacco), Coke & Petroleum, and Pharmaceuticals. Secondly, this study relies on cross-sectional tactics to gather the data. The future researcher may use a longitudinal study to collect better knowledge of industry employees. Third, the limitation is associated with the fact that this research study did not contemplate non-academic literature, including blogs, technical reports, and newspapers such as “Grey literature”. Hence, future researchers are suggested to incredulous such limitations and progress the literature. Fourth, current research perceives that government support and regulations concerning blockchain technology are favorable, and all regulatory context supports using blockchain technology in supply chain parties. In Pakistan, India, and many other countries, no certified frameworks regulate and administer blockchain technology and its applications. Hence, future researchers can practice this study in regions where countries have fully developed and administered blockchain technologies. Fifth, this study assumes that the firms have already adopted blockchain technology in their supply chain operations. However, the level of blockchain technology implementation and its impact on firm performance may vary across organizations. Future research could examine blockchain technology's adoption and utilization levels and their implications for firm performance. Sixth, this study used trust as a mediating variable between supply chain parameters' impact on firm performance. Future research could investigate potential mediating variables such as operational efficiency, customer satisfaction, and technology innovation to test the underlying mechanisms. Lastly, this study focused on blockchain technology to improve the supply chain process toward firm performance. Future studies will compare the effects of different technologies and approach to supply chain management, including blockchain, traditional systems, and hybrid models, enabling a better assessment of blockchain technology's specific advantages and limitations in improving supply chain performance.

## Funding

This work received financial support from the 10.13039/501100001809National Natural Science Foundation of China under grant number 72074014.

## Data availability statement

Data will be made available on request.

## CRediT authorship contribution statement

**Muhammad Farrukh Shahzad:** Writing – review & editing, Writing – original draft, Validation, Software, Project administration, Methodology, Formal analysis, Conceptualization, Resources, Visualization. **Shuo Xu:** Supervision, Investigation, Conceptualization, Funding acquisition, Writing – original draft. **Rimsha Baheer:** Writing – original draft, Resources, Methodology, Validation. **Waleed Ahmad:** Investigation, Validation, Writing – review & editing.

## Declaration of competing interest

There is no conflict of interest.
