# Corrigendum to “Unveiling the role of supply chain parameters approved by blockchain technology towards firm performance through trust: The moderating role of government support” [Heliyon Volume 9, Issue 11, November 2023, Article e21831]

**DOI:** 10.1016/j.heliyon.2025.e43359

**Published:** 2025-04-30

**Authors:** Muhammad Farrukh Shahzad, Shuo Xu, Rimsha Baheer, Waleed Ahmad

**Affiliations:** a College of Economics and Management, Beijing University of Technology, Beijing, 100124, PR China; b Institute of Business & Management, University of Engineering and Technology, Lahore, 54000, Pakistan

In the original published version of this article, the ethics and consent statement were missing. The article has been updated to include the missing details.

The corrected version of ethics and consent statement can be found below:

**Ethics approval and consent to participate**

This study was evaluated by Beijing University of Technology, China dated 07/03/2023 under financial project number: 72074014. This study was conducted using an online survey method. All participants were informed about the study's purpose, and their participation was entirely voluntary. Informed consent was obtained from all participants before proceeding with the survey.

The authors apologize for the errors.

## Declaration of competing interest

There is no conflict of interest.

