# Supplementary material for: Unveiling the role of supply chain parameters approved by blockchain technology towards firm performance through trust: The moderating role of government support
Source: Heliyon. 2023 Nov 4;9(11):e21831. doi: 10.1016/j.heliyon.2023.e21831 (PMC10663855; doi:10.1016/j.heliyon.2023.e21831)
Supplement: Multimedia component 1 [file mmc1.docx]

**Unveiling the role of supply chain parameters approved by blockchain technology towards firm performance through trust: The moderating role of government support**

**Note:** I am conducting research as a student of PhD in Management Sciences and Engineering School of Economics and Management, Beijing University of Technology, Beijing China. I am conducting research on **“Unveiling the role of supply chain parameters approved by blockchain technology towards firm performance through trust: The moderating role of government support”.** You are requested to spare your precious time to complete this survey. Your specific answers will be completely anonymous & confidential, but your views, in combination with those of others, are extremely important. Your cooperation is highly appreciated. Thanks once again for your time and cooperation.

**Section-1 Personal Memoranda**

1. Name (Optional)------------------------------------------------------------------------
2. Phone No (Optional)-------------------------------------------------------------------
3. Email Address (Optional)---------------------------------------------------------------
4. Designation-------------------------------------------------------------------------------
5. Department--------------------------------------------------------------------------------
6. Experience of working--------------------------------------------------------------------
7. Qualification:

- Post-graduate-----------------------------------------------------------------------
- Professional Graduate--------------------------------------------------------------
- Graduate-----------------------------------------------------------------------------
- Any other----------------------------------------------------------------------------

1. Gender of Respondent:

- Male------------------------------------
- Female---------------------------------

**Section – 2 (Please reply all the research questions.)**

1. **Supply Chain Alignment**

| **Encircle only one number from 1-5 that indicates your disagreement or agreement** | | **Strongly**  **disagree** | **Disagree** | **Neutral** | **Agree** | **Strongly agree** |
| --- | --- | --- | --- | --- | --- | --- |
| SCAL1 | Specific formal efforts to share supply chain vision/strategy throughout the organization. |  |  |  |  |  |
| SCAL2 | Specific formal efforts to coordinate objectives throughout the organization. |  |  |  |  |  |
| SCAL3 | Specific formal efforts to achieve consistency in operating procedures throughout the organization. |  |  |  |  |  |
| SCAL4 | Lays down roles, tasks, and responsibilities clearly for suppliers and customers |  |  |  |  |  |
| SCAL5 | Exchanges information and knowledge freely with vendors and customers |  |  |  |  |  |
| SCAL6 | Equitably shares risks, costs, and gains of improvement initiatives |  |  |  |  |  |

1. **Supply Chain Agility**

| **Encircle only one number from 1-5 that indicates your disagreement or agreement** | | **Strongly**  **disagree** | **Disagree** | **Neutral** | **Agree** | **Strongly agree** |
| --- | --- | --- | --- | --- | --- | --- |
| SCAG1 | Works hard to promote the flow of information with its suppliers and Customers. |  |  |  |  |  |
| SCAG2 | Works hard to develop collaborative relationships with suppliers. |  |  |  |  |  |
| SCAG3 | Builds inventory buffers by maintaining a stockpile of inexpensive but key components. |  |  |  |  |  |
| SCAG4 | Has a dependable logistics system or partner? |  |  |  |  |  |
| SCAG5 | Draws up contingency plans and develops crisis management teams. |  |  |  |  |  |

1. **Supply Chain Adaptability**

| **Encircle only one number from 1-5 that indicates your disagreement or agreement** | | **Strongly**  **disagree** | **Disagree** | **Neutral** | **Agree** | **Strongly agree** |
| --- | --- | --- | --- | --- | --- | --- |
| SCAD1 | Monitors economies all over the world to spot new supply bases and markets. |  |  |  |  |  |
| SCAD2 | Uses intermediaries to develop fresh suppliers and logistics infrastructure. |  |  |  |  |  |
| SCAD3 | Evaluates needs of ultimate consumers not just immediate customers. |  |  |  |  |  |
| SCAD4 | Creates flexible product designs. |  |  |  |  |  |
| SCAD5 | Determines where the company’s products stand in terms of technology cycles and product life cycles. |  |  |  |  |  |

1. **Trust**

| **Encircle only one number from 1-5 that indicates your disagreement or agreement** | | **Strongly**  **disagree** | **Disagree** | **Neutral** | **Agree** | **Strongly agree** |
| --- | --- | --- | --- | --- | --- | --- |
| T1 | This supplier is trustworthy keeps promises it makes to our firm. |  |  |  |  |  |
| T2 | We believe the information that this vendor provides us. |  |  |  |  |  |
| T3 | This supplier is genuinely concerned that our business succeeds. |  |  |  |  |  |
| T4 | When making important decisions, this supplier considers our welfare as well as its own. |  |  |  |  |  |
| T5 | We trust this supplier keeps our best interests in mind. |  |  |  |  |  |

1. **Government Support**

| **Encircle only one number from 1-5 that indicates your disagreement or agreement** | | **Strongly**  **disagree** | **Disagree** | **Neutral** | **Agree** | **Strongly agree** |
| --- | --- | --- | --- | --- | --- | --- |
| GS1 | Government supports of blockchain implementation would provide an incentive to use in supply chain. |  |  |  |  |  |
| GS2 | Government regulations and monitoring would reduce the risks associated with using blockchain in supply chain. |  |  |  |  |  |
| GS3 | The government should support or be responsible for regulating technology influence our firm performance. |  |  |  |  |  |
| GS4 | Regulations and government insurance should exist to protect the users and trust of people. |  |  |  |  |  |

1. **Firm Performance**

| **Encircle only one number from 1-5 that indicates your disagreement or agreement** | | **Strongly**  **disagree** | **Disagree** | **Neutral** | **Agree** | **Strongly agree** |
| --- | --- | --- | --- | --- | --- | --- |
| Please indicate the choice that accurately reflects your firm’s overall performance. | | | | | | |
| FP1 | Market share |  |  |  |  |  |
| FP2 | Return on investment |  |  |  |  |  |
| FP3 | The growth of market share |  |  |  |  |  |
| FP4 | Growth in return on investment |  |  |  |  |  |
| FP5 | Profit margin on sales |  |  |  |  |  |
| FP6 | Overall competitive position |  |  |  |  |  |
